# Frequency-Dependent Changes in Interhemispheric Functional Connectivity Measured by Resting-State fMRI in Children With Idiopathic Generalized Epilepsy

**DOI:** 10.3389/fneur.2020.00645

**Published:** 2020-07-29

**Authors:** Lin Jiang, Xuejin Ma, Shiguang Li, Hongjian Luo, Guoming Zhang, Yanan Wang, Tijiang Zhang

**Affiliations:** ^1^Department of Radiology, The Third Affiliated Hospital of Zunyi Medical University, Zunyi, China; ^2^Department of Radiology, Medical Imaging Center of Guizhou Province, Affiliated Hospital of Zunyi Medical University, Zunyi, China

**Keywords:** fMRI, interhemispheric, functional connectivity, frequency-dependent, IGE

## Abstract

Epilepsy is associated with abnormal spatiotemporal changes in resting-state brain connectivity, but how these changes are characterized in interhemispheric coupling remains unclear. This study aimed to characterize frequency-dependent alterations in voxel-wise mirrored homotopic connectivity (VMHC) measured by resting-state functional magnetic resonance imaging (rs-fMRI) in children with idiopathic generalized epilepsy (IGE). Rs-fMRI data were collected in 21 children with IGE and 22 demographically matched children with typical development. We used three resting-state frequency bands (full, 0.01–0.08 Hz; slow-4, 0.027–0.073 Hz; slow-5, 0.01–0.027 Hz) to compute VMHC and locate the significant foci. Voxel-wise *p* <0.001 and cluster-level *p* <0.05 cluster-level family-wise error correction was applied. In between-group comparisons, we identified that the full and higher frequency (slow-4) bands showed similar reductions in VMHC including Rolandic operculum, putamen, superior frontal, lateral parietal, middle cingulate, and precuneus in children with IGE. In the lower frequency band (slow-5), we identified specific reductions in VMHC in orbitofrontal and middle temporal gyri in children with IGE. Further analyses on main effects and interaction between group and frequency band suggested significant frequency-dependent changes in VMHC, and no significant interaction was found. The results were generally similar with global brain signal regression. Additional association analysis showed that VMHC in the putamen within the full and slow-4 bands was significantly positively correlated with chronological age in children with IGE, and the same analysis was non-significant in the controls; VMHC in the medial prefrontal region in the slow-4 band was significantly positively correlated with IQ performance sub-score. Our findings suggest that IGE children show frequency-dependent changes in interhemispheric integration that spans regions and systems involving cortical-subcortical, language, and visuomotor processing. Decreased functional coupling within the dorsal striatum may reflect atypical development in children with IGE.

## Introduction

Idiopathic generalized epilepsy (IGE) is characterized by generalized spike-wave discharges on normal electroencephalography (EEG) and accounts for 15–20% of all epilepsies (1), with no identifiable causes other than genetic factors (2, 3). A majority of IGE originates in childhood and primarily encompasses three subgroups, namely juvenile absence epilepsy, juvenile myoclonic epilepsy, and generalized tonic-clonic seizures in individuals under 18 years old (4, 5).

Because there are no obvious abnormalities on conventional MRI, advanced neuroimaging, including structural and functional magnetic resonance imaging (fMRI), is a promising tool for the investigation of IGE (3, 6, 7). For example, results from structural imaging have reported abnormalities in the medial/orbitofrontal cortex, cingulate cortex, precuneus, and thalamus in patients with IGE (6, 8–11). Resting-state fMRI (rs-fMRI) measured with low-frequency (typically <0.1 Hz) blood oxygenation level-dependent signals are crucial is understanding brain function under healthy or disease states due to its ability to reveal the spatiotemporal structure of spontaneous activity in the human brain (12, 13).

Functional connectivity (FC), which has been widely applied in fMRI and confirmed to be associated with abnormal discharges on EEG, quantifies temporal correlations between brain regions to interrogate the direct or indirect interregional interactions (2, 6, 14). Studies have demonstrated that FC within some resting-state networks such as self-referential, somatosensory, visual, and auditory networks and the classic default-mode and dorsal attention networks is disrupted in patients with IGE (6, 15, 16). Moreover, as some recent publications of the International League Against Epilepsy (ILAE) on revised terminology of seizures and epilepsies have reflected, IGE originates from a local region within a single cerebral hemisphere and rapidly spreads to bilateral networks (17). A method named voxel-mirrored homotopic connectivity (VMHC) has been widely used in various mental illnesses like schizophrenia and epilepsy to acquire information on interhemispheric communication (18, 19). Also, Ji et al. used VMHC and reported that interhemispheric FC between the bilateral cuneus and anterior cingulate cortex increased and that between the bilateral olfactory cortex, inferior frontal gyrus, supramarginal gyrus, and temporal pole decreased in patients with generalized tonic-clonic seizures (20).

However, these studies focused on FC within a typical low-frequency range (0.01–0.08 Hz), which did not consider temporal differences in intrinsic activity. Recent studies have shown that the intrinsic activity within different frequency bands differs and that frequency-dependent changes occur in a variety of brain disorders (21). Therefore, in this study, we tested whether FC changes between bilateral hemispheric regions are frequency-dependent.

## Materials and Methods

### Participants

Twenty-one children with IGE (13 males/8 females, 11.48 ± 3.46 years), according to ILAE criteria (22) were consecutively enrolled from the Affiliated Hospital of Zunyi Medical University, Zunyi, China (from December 2013 to January 2018), and 22 age-, sex-, and education-matched healthy controls (9 males/13 females, 12.09 ± 2.93 years) were also recruited. The inclusion criteria were as follows: (a) manifestation of IGE; (b) presence of diffuse poly-spike-wave complex on patient's scalp EEG; (c) no evidence of a cause of secondary IGE, such as tumor, trauma, or intracranial infection; (d) no focal abnormality with conventional MRI; (e) right-handedness; (f) 7–18 years old. The exclusion criteria were as follows: (a) had a contraindication for MRI; (b) had a history of neurological disorders other than epilepsy; (c) fell asleep during rs-fMRI scanning; (d) head motion exceeded ±1 mm or ± 1°. There were no significant difference in age (*p* = 0.53, *p* > 0.05, two-sample independent *t*-test), sex (*p* = 0.23, *p* > 0.05, Chi-square test) between the IGE and control groups. The significance of *p* value is set at a level of 0.05. This study was approved by the medical ethics committee of the Affiliated Hospital of Zunyi Medical University, and written informed consent was obtained from each participant before evaluation.

### Data Acquisition

Rs-fMRI data were acquired with a GE 3.0-T (HDxt, GE Healthcare) scanner with a standard head coil in the Department of Radiology, Affiliated Hospital of Zunyi Medical University. For each subject, functional images were acquired by using a single-shot, gradient-recalled echo-planar imaging sequence (repetition time = 2,000 ms, echo time = 30 ms, and flip angle = 90°), containing 30 transverse sections (field of view = 240 × 240 mm^2^, slice thickness = 5 mm with no gap, and voxel size = 3.75 × 3.75 × 4 mm^3^), resulting in a total imaging time of 413 s. The subjects were instructed to rest with eyes closed but awake and not think of anything in particular. Three-dimensional T1-weighted imaging (3D-T1WI) images were acquired by using a 3D-T1WI brain volume imaging (BRAVO) sequence (repetition time = 1,900 ms, echo time = 2.1 ms, inversion time = 900 ms, flip angle = 9°, slice thickness = 1.00 mm, and matrix = 256 × 256), yielding 160 axial slices with an in-plane resolution of 1.0 mm × 1.0 mm.

### Data Processing

The preprocessing was performed using the Data Processing Assistant for Resting-State fMRI (DPARSF) (23) and statistical parametric mapping (SPM8, http://wwwfil.ion.ucl.ac.uk/spm). The first 10 volumes were removed to avoid instability of the machine, and the remaining volumes were corrected for temporal differences and head motion (Friston 24-parameter model). Then, the corrected volumes were coregistered to individual T1 images. The T1 images were segmented into white matter, gray matter, and cerebrospinal fluid and normalized to Montreal Neurologic Institute space using 24-parameter transformation and non-linear deformations. The functional images were then warped with the same parameters and resampled at a resolution of 3 × 3 × 3 mm^3^. After spatial normalization, the volumes were detrended. We then regressed the nuisance covariates containing head motion estimates, averaged time series in white matter and cerebrospinal fluid, with and without global signals. Based on an earlier study (13), we calculated three low-frequency bands: full band (0.01–0.08 Hz), slow-5 (0.01–0.027 Hz), and slow-4 (0.027–0.073 Hz) to examine the frequency-specific brain activity changes in the children with IGE.

For head motion estimates, no subjects were excluded with a criterion of >2-mm maximum displacement or >2° rotation. We also calculated micro head motion with frame-wise displacement (FD). Three FD parameters, namely mean FD Power (IGE 0.245 ± 0.148, HC 0.214 ± 0.212, *t* = −0.556, *p* = 0.581) (24), mean FD Jenkinson (IGE 0.122 ± 0.075, HC 0.116 ± 0.127, *t* = −0.188, *p* = 0.852) (25), and mean FD VanDijk (IGE 0.064 ± 0.045, HC 0.051 ± 0.043, *t* = −0.992, *p* = 0.327) (26), were calculated, and there was no significant difference between the IGE and HC groups.

### VMHC

The calculation of VMHC has been detailed in Zuo et al. (27) Briefly, the “clean” functional volumes of the abovementioned low-frequency filtering were first registered to a high-resolution left-right symmetrical anatomical template. Then, a high-dimensional voxel-level interhemispheric FC map, also termed the VMHC map, was calculated. The individual VMHC map was Z-transformed and spatially smoothed (4-mm full-width at half-maximum) to improve normality and the signal-to-noise ratio (20).

### Statistical Analysis

Independent two-sample *t*-tests were used to analyze between-group differences on VMHC across the three frequency bands (full, slow-4, and slow-5). To determine main effects of frequency band and group and their interaction, we also performed a 2 (groups: IGE and HC) × 2 (frequency bands: slow-4 and slow-5) two-way analysis of variance (ANOVA). The mean FD Power was regressed out as confounding covariates. The results were voxel-wise *p* <0.001, cluster level *p* <0.05, family-wise error (FWE) cluster-level corrected. Moreover, we performed association analyses between the VMHC values in all clusters showing significant differences and behavioral variables in the IGE children.

## Results

### Demographics

Table 1 shows the demographics for all subjects. There were no significant difference in age (*p* = 0.53, *p* > 0.05, two-sample independent *t*-test), sex (*p* = 0.23) and education (*p* = 0.08) (*p* > 0.05, Chi-square test) between the IGE and control groups (Table 1).

**Table 1:** Demographic and clinical results.

**Items**	**IGE (*n* = 21)**	**Controls (*n* = 22)**	***t***	***p***
Sex (M/F)	13/8^*^	9/13^*^	−1.38	0.23^‡^
Age (y)	11.48 ± 3.46	12.09 ± 2.93	−0.63	0.53^†^
Education (y)	4.76 ± 3.22	6.55 ± 3.21	−1.815	0.08^†^
Handedness (R/L)	21/0^*^	22/0^*^		
Duration (y)	3.07 ± 2.51			
IQ overall	81.00 ± 19.00			
IQ language	83.00 ± 21.00			
IQ performance	82.00 ± 18.00			

* *Data are the number of subjects*.

† *Two-sample t-test was used*.

‡ *χ^2^ test was used*.

### Between-Group Comparison of VMHC

**Figure 1:**
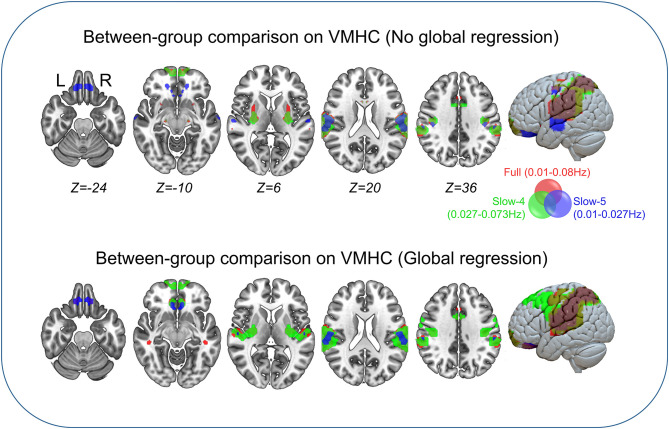
Between-group comparisons on VMHC. The three color labels represent statistically significant between-group differences, i.e., significantly decreased VMHC between the IGE and HC groups under the three frequency bands (red, typical band: 0.01–0.08 Hz; green, slow-4: 0.027–0.73 Hz; blue, slow-5: 0.01–0.027 Hz). The composed map reflects the topographical distribution of frequency-specific changes. These results were voxel-wise *p* <0.001, cluster level *p* <0.05, family-wise error (FWE) cluster-level corrected.

Between-group comparisons of VMHC are shown in Figure 1, Tables 2–**4**. In the conventional low-frequency band (0.01–0.08 Hz), we found significantly decreased VMHC in Rolandic operculum, supramarginal gyrus, putamen, middle orbitofrontal, superior parietal, and middle cingulate regions in children with IGE (Figure 1, Table 2).

**Table 2:** Between-group comparison on VMHC in the full band.

**Brain regions**	**MNI coordinates**			**Voxels**	***T*-values**
	**X**	**Y**	**Z**		
**No global regression**
Rolandic operculum	±57	−18	15	990	−4.365
Supramarginal gyrus	±60	−33	42	990	−4.342
Supramarginal gyrus	±69	−36	24	990	−4.322
Putamen	±27	−12	6	179	−4.005
Middle frontal gyrus, orbital part	±6	63	−9	70	−3.761
Superior parietal	±12	−72	51	55	−3.728
Middle cingulate cortex	±3	18	30	42	−3.671
**With global regression**
Supramarginal gyrus	±60	−33	42	1,387	−5.479
Superior parietal	±42	−54	57	1,387	−5.216
Supramarginal gyrus	±63	−27	21	1,387	−4.801
Anterior cingulate cortex	±9	33	−9	71	−3.815
Middle frontal gyrus, orbital part	±6	60	−12	50	−3.679
Middle cingulate cortex	±3	18	30	42	−3.589

*Regions showing decreased interhemispheric connectivity in children with IGE vs. controls. MNI, Montreal Neurologic Institute*.

We then decomposed this typical frequency band into two sub-bands, slow-4 (0.027–0.073 Hz) and slow-5 (0.01–0.0273 Hz). As expected, we identified significant between-group differences in inferior parietal, supramarginal, putamen, superior frontal, and precuneus regions that were similar to those in the conventional band, with significantly decreased VMHC (Figure 1, Table 3).

**Table 3:** Between-group comparison on VMHC in the slow-4 band.

**Brain regions**	**MNI coordinates**			**Voxels**	***T*-values**
	**X**	**Y**	**Z**		
**No global regression**
Inferior parietal gyrus	±45	−48	54	1,109	−5.123
Supramarginal gyrus	±69	−33	24	1,109	−4.728
Supramarginal gyrus	±57	−36	42	1,109	−4.584
Putamen	±27	−15	6	172	−4.305
Superior frontal gyrus	±9	60	−9	88	−4.204
Precuneus	±12	−69	51	63	−3.872
Middle cingulate	±3	15	30	50	−3.756
**With global regression**
Inferior parietal gyrus	±42	−54	57	1,709	−6.116
Supramarginal gyrus	±57	−33	42	1,709	−5.493
Supramarginal gyrus	±69	−33	24	1,709	−5.277
Superior frontal gyrus	±6	60	−9	72	−4.541
Anterior cingulate	±9	33	−9	59	−4.101
Middle frontal gyrus	±36	21	48	64	−3.999
Middle cingulate	±3	15	30	48	−3.711
Supplementary motor area	±3	−3	48	48	−3.144
Superior frontal gyrus	±18	36	54	45	−3.456

*Regions showing decreased interhemispheric connectivity in children with IGE vs. controls. MNI, Montreal Neurologic Institute*.

We next examined the slow-5 band and found that the children with IGE showed significantly decreased VMHC in middle orbital, lateral temporal, lateral parietal, and postcentral regions (Figure 1, Table 4). These results were generally consistent with the results with global brain signal regression (Figure 1, Tables 2–4) and reported at voxel-wise *p* <0.001 and cluster-level *p* <0.05, FWE cluster corrected.

**Table 4:** Between-group comparison on VMHC in the slow-5 band.

**Brain regions**	**MNI coordinates**			**Voxels**	***T*-values**
	**X**	**Y**	**Z**		
**No global regression**
Middle orbital gyrus	±12	36	−3	103	−3.153
Superior temporal gyrus	±63	−27	18	285	−4.068
Middle temporal gyrus	±60	−9	−3	285	−3.665
Supramarginal gyrus	±60	−33	42	285	−2.835
Superior parietal lobule	±33	−63	63	92	−3.694
Inferior parietal lobule	±45	−42	51	92	−3.240
Postcentral gyrus	±42	−24	39	61	−3.608
**With global regression**
Postcentral gyrus	±60	−24	24	178	−4.297
Supramarginal gyrus	±60	−33	42	178	−3.207
Superior temporal gyrus	±57	−15	3	178	−2.929
Superior parietal lobule	±39	−54	66	114	−3.969
Rectus gyrus	±9	36	−24	115	−3.923

*Regions showing decreased interhemispheric connectivity in children with IGE vs. controls. MNI, Montreal Neurologic Institute*.

### Main Effects and Interaction Between Group and Frequency Band

**Figure 2:**
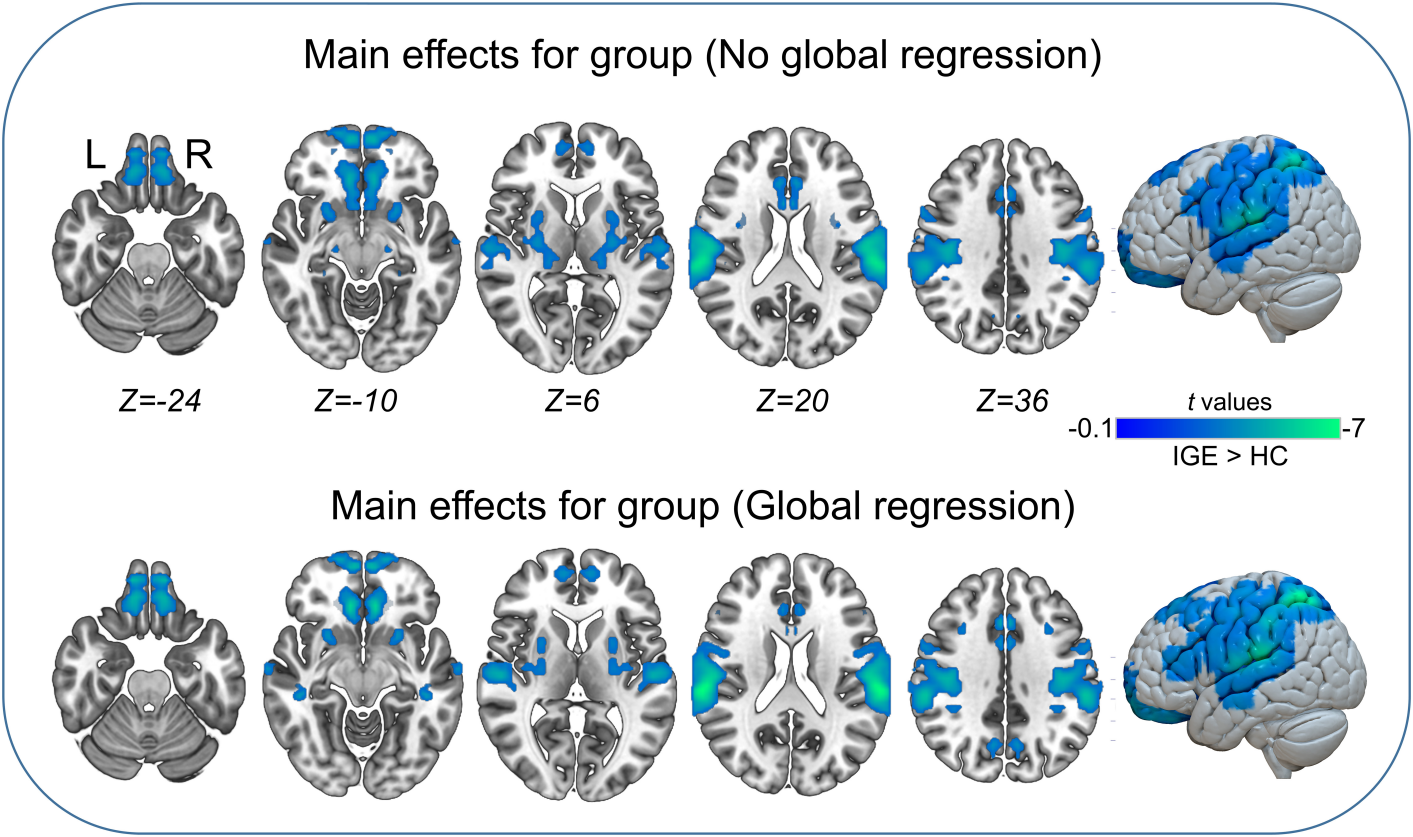
Main effects for group. Only significantly reduced VMHC was found (cold colorbar, IGE < HC). The results were voxel-wise *p* <0.001, cluster level *p* <0.05, family-wise error (FWE) cluster-level corrected. L, left; R, right.

**Figure 3:**
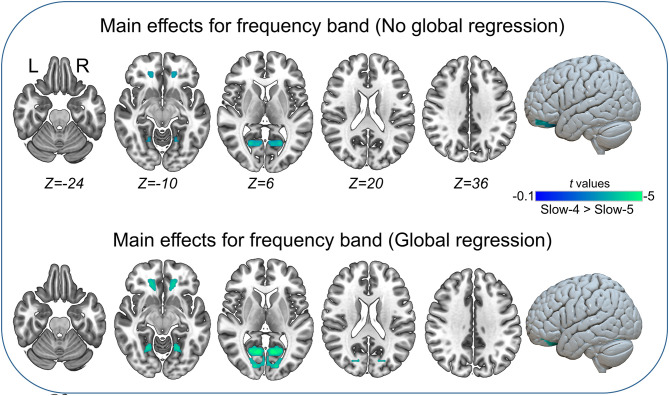
Main effects for frequency band. Only significantly reduced VMHC was found (cold colorbar, slow-4 < slow-5). The results were voxel-wise *p* <0.001, cluster level *p* <0.05, family-wise error (FWE) cluster-level corrected. L, left; R, right.

The main effects for group showed that the IGE group had significant decreases on VMHC in supramarginal, superior parietal, postcentral, anterior cingulate, rectus, thalamus, putamen, Rolandic operculum, and medial superior frontal regions (Figure 2, Table 5). The main effects for frequency band showed that the slow-4 band had significant decreases on VMHC in lingual, calcarine, and middle orbitofrontal regions. These results were generally consistent with the results with global brain signal regression (Figure 3, Table 6) and reported at voxel-wise *p* <0.001 and cluster-level *p* <0.05, FWE cluster corrected. No significant interaction was found between the frequency band and the group.

**Table 5:** Main effects for group (IGE > HC) on VMHC.

**Brain regions**	**MNI coordinates**			**Voxels**	***T*-values**
	**X**	**Y**	**Z**		
**No global regression**
SupraMarginal gyrus	±63	−27	18	1,872	−5.899
Superior parietal gyrus	±42	−54	57	1,872	−5.462
Postcentral gyrus	±42	−15	42	1,872	−5.102
Anterior cingulate	±9	36	−6	558	−4.728
Gyrus rectus	±9	39	−27	558	−4.348
Anterior cingulate	±3	15	24	134	−4.823
Thalamus	±18	−18	−3	309	−4.614
Putamen	±24	9	−9	309	−3.537
Rolandic operculum	±33	3	15	309	−3.659
Superior frontal gyrus, medial	±12	27	63	125	−3.946
**With global regression**
Superior parietal gyrus	±42	−54	60	1,967	−6.274
Supramarginal gyrus	±63	−27	18	1,967	−6.020
Supramarginal gyrus	±60	−33	42	1,967	−5.649
Anterior cingulate	±9	33	−9	536	−5.081
Superior frontal gyrus, medial	±9	54	6	536	−3.393
Middle cingulate	±3	21	30	112	−4.388
Superior frontal gyrus, medial	±12	27	63	176	−4.366
Superior parietal gyrus	±12	−72	51	83	−4.119
Inferior frontal gyrus, triangular part	±51	27	15	48	−3.892
Insula	±33	0	15	133	−3.354
Middle frontal gyrus	±36	21	48	61	−3.677

*Regions showing decreased interhemispheric connectivity in children with IGE vs. controls. MNI, Montreal Neurologic Institute*.

**Table 6:** Main effects for frequency band (slow-4 > slow-5) on VMHC.

**Brain regions**	**MNI coordinates**			**Voxels**	***T*-values**
	**X**	**Y**	**Z**		
**No global regression**
Lingual gyrus	±3	−69	−3	208	−3.610
Calcarine fissure and surrounding cortex	±21	−66	15	208	−3.568
Middle frontal gyrus, orbital part	±18	39	−9	49	−3.045
**With global regression**
Lingual gyrus	±9	−66	3	311	−4.162
Calcarine fissure and surrounding cortex	±15	−87	6	311	−3.249
Middle frontal gyrus, orbital part	±15	39	−6	68	−3.320

*Regions showing decreased interhemispheric connectivity in children with IGE vs. controls. MNI, Montreal Neurologic Institute*.

### Brain-Behavior Association

**Figure 4:**
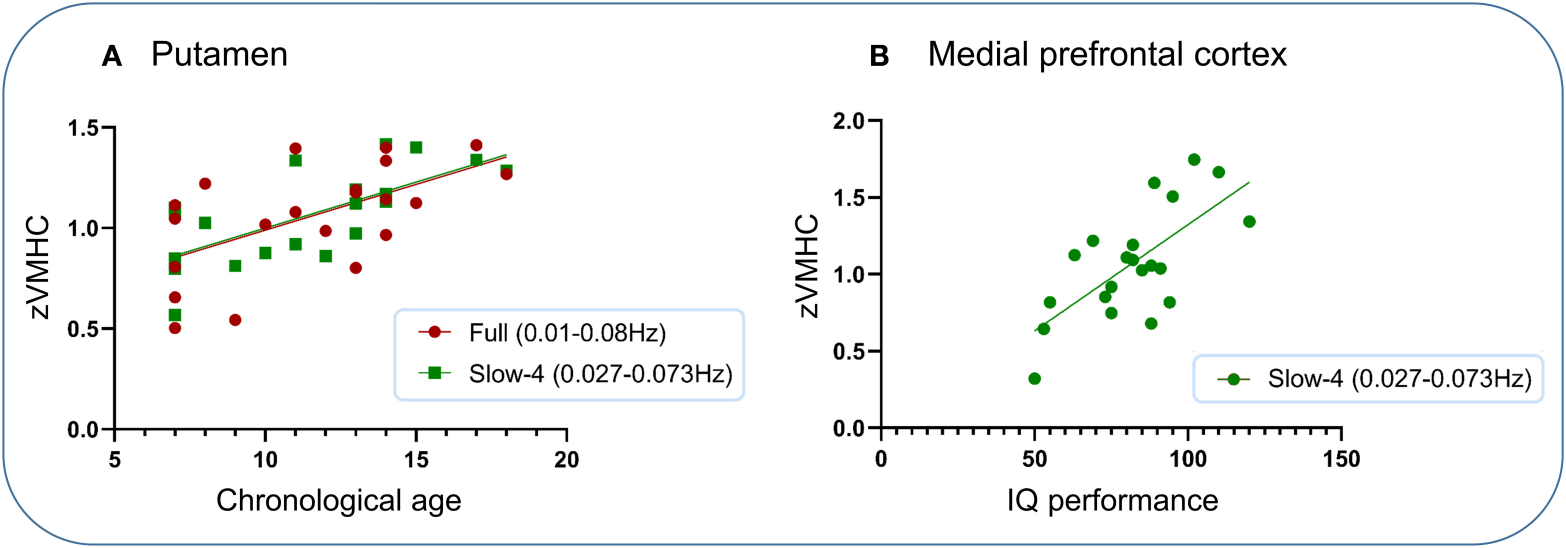
Association analysis. Significantly positive associations were found between VMHC within the putamen under two frequency bands (full and slow-4) and chronological age and between VMHC within the medial prefrontal cortex and IQ performance subscore. **(A)** Putamen. **(B)** Medial prefrontal cortex.

Significantly positive associations were found between VMHC within the putamen under two frequency bands (full and slow-4) and chronological age and between VMHC within the medial prefrontal cortex and IQ performance subscore (Figure 4). No other significant associations were found.

## Discussion

We investigated interhemispheric FC across different frequency bands with rs-fMRI in children with IGE. Similar reductions on VMHC in children with IGE were found in the Rolandic operculum, putamen, superior frontal, lateral tempo-parietal, middle cingulate, and precuneus, in both the full and slow-4 frequency ranges. Meanwhile, specific reductions in orbitofrontal and middle temporal gyri in children with IGE were found in a lower frequency band (slow-5). Further analyses on main effects and interaction between group and frequency band suggested significant frequency-dependent changes in VMHC, and no significant interaction was found. The results were generally similar with global brain signal regression. Additional association analysis showed that VMHC in the putamen within the full and slow-4 bands was significantly positively correlated with chronological age in children with IGE, and the same analysis was non-significant in the controls; VMHC in the medial prefrontal region in the slow-4 band was significantly positively correlated with IQ performance sub-score. Our findings suggest that IGE children show frequency-dependent changes in interhemispheric integration that spans regions and systems involving cortical-subcortical, language, and visuomotor processing. Decreased functional coupling within the dorsal striatum may reflect atypical development in children with IGE.

Our results from both the between-group comparisons and main effects are generally consistent with previous reports (20, 28–34), especially an earlier report that exclusively investigated VMHC changes in the typical frequency band (20). In that study, the authors found both increased and decreased interhemispheric FC in widespread regions, including increased VMHC in cuneus and anterior cingulate cortices, and decreased VMHC in olfactory, inferior frontal, supramarginal, and temporal regions (20). Although our study did not identify a significant increase in VMHC, the anatomical configuration of the brain regions involved is generally consistent with that study.

IGE has been conceptualized to originate at one point within a unilateral hemisphere and propagate bilaterally (17). FC between bilateral hemispheres might be linked to the interhemispheric communication that can reflect the integrity of brain function (20, 35). We first identified significant reductions in VMHC including Rolandic operculum, putamen, superior frontal, lateral tempo-parietal, middle cingulate, and precuneus in children with IGE in the conventional frequency band (full band). These reductions, especially in the dorsal striatum, were generally replicated in the higher frequencies of the slow-4 band, suggesting that the between-group differences of the conventional band (full) are largely contributed by the slow-4 band. Across the three frequency bands, the statistical differences between groups converge to the lateral temporoparietal areas. Meanwhile, unique reductions in orbitofrontal and middle temporal gyri in children with IGE were found in a lower frequency band (slow-5).

The putamen and superior frontal gyrus have been suggested to be involved in motor-related behavior (36), linguistic processing (37), and auditory processing (38), and some researchers have suggested that the superior temporal gyrus may be involved in theory of mind ability (39). At the same time, patients with IGE have been demonstrated to have cognitive impairments and behavioral deficits, and related functions, such as memory, attention, and linguistic function, were weakened (6, 20, 21, 34, 37, 40). Together with the significant associations between VMHC in the putamen and age as found in this study, therefore, it is reasonable to believe that decreased FC in the abovementioned regions might be correlated to the functional deficits in patients with IGE.

The default network, which mainly comprises the posterior cingulate cortex/precuneus and the medial prefrontal, is proposed to be the biological basis of self-information, emotion, and social skills (41, 42). Decreased FC within the default network has been associated with consciousness impairments and behavioral deficits in individuals with IGE (6, 15, 37, 40). Our results showing decreased VMHC within the orbitofrontal, medial prefrontal, and posterior cingulate cortices may have led to impairments in reward processing and decision making of the patients, resulting in a decline in their ability to learn new things (43, 44). One explanation is that decreased VMHC of the medial prefrontal/orbitofrontal regions involved in emotion management and decision making may lead to executive dysfunctions in those with IGE. In line with our results, both increased and decreased FC were also observed within the default network (15, 16, 34). Moreover, we observed decreased VMHC in the putamen, orbitofrontal, visual, lateral temporoparietal, and somatomotor regions, which might be associated with reductions in motor management (6), especially the experience of visual aura (45) and myoclonic jerks (40) in patients with IGE.

Based on previous studies (7, 11, 20), we investigated interhemispheric FC in different frequency bands and observed that the interhemispheric communication changes measured in rs-fMRI in individuals with IGE were frequency-dependent. Significant interhemispheric changes were localized in the bilateral OFC and lingual gyrus in the slow-5 band, while the changes in the slow-4 band were different, including the bilateral putamen and bilateral superior temporal gyrus. These findings were in line with previous studies (13) that showed that the amplitude of low-frequency fluctuations in the slow-4 band is sensitive to subcortical activity (more robust in basal ganglia), whereas the intensity of amplitude of low-frequency fluctuations in the slow-5 band was more dominant within the cortical regions, such as the medial prefrontal (13, 46). Therefore, different interhemispheric FC change distributions between the full frequency band and the slow-5 band may indicate the higher sensitivity of the slow-5 band for detecting abnormal low-frequency fluctuation changes in individuals with IGE. Thus, our findings add to the growing body of evidence that such frequency-specific effects linked with low-frequency fluctuations may be crucial to the origination and development of brain functions and enable a better understanding of the coordinated brain activity in patients with brain disorders (46).

We also analyzed the potentially different statistical results in the case of global brain signal regression. Surprisingly, the global brain signal regression and non-global brain signal regression showed very similar results, which seems to be different from what we originally thought: that epilepsy is accompanied by abnormal brain signals. Some controversy exists regarding the regression of global signals, with authors reporting that global regression may introduce false-negative correlations and eliminate positive correlations (35, 37, 47). One study conducted in rats after dopamine loss found the presence of negative correlations between nodes in the brain (48). In addition, Fox et al. (49) suggested that negative correlations existed as a biological basis before the global regression and may play a crucial role in differentiating neuronal processes of opposite or competing activities, while the widely distributed global signals of gray matter might obscure potential neuroanatomical relationships. Therefore, the regression step contributes to removing the confounding factors and clearly presents the characteristics of the anticorrelation network (50).

There are several limitations to our study. First, though the IGE children showed good coordination and head movement control during the acquisition of rs-fMRI, we did not conduct simultaneous EEG monitoring at the same time, and we cannot rule out abnormal interictal discharges during this period, but we estimate that this possibility is relatively small. Second, the IGE children included in this study were more or less antiepileptic drug-treated depending on the course of the disease. Therefore, it is difficult to elaborate on the influence of drugs on connectivity results. Third, the sample size of our study was relatively small, and different IGE subsyndromes may have different abnormalities in brain structure and FC. Therefore, future studies should expand the sample size and investigate different subtypes of epileptic patients.

## Conclusion

Our findings suggest that IGE children show frequency-dependent changes in interhemispheric integration that spans regions and systems involving cortical-subcortical, language, and visuomotor processing. Decreased functional coupling within the dorsal striatum may reflect atypical development in children with IGE.

## Data Availability Statement

The raw data supporting the conclusions of this article will be made available by the authors, without undue reservation, to any qualified researcher.

## Ethics Statement

The studies involving human participants were reviewed and approved by Medical Ethics Committee of Zunyi Medical University. Written informed consent to participate in this study was provided by the participants' legal guardian/next of kin. Written informed consent was obtained from the individual(s), and minor(s)' legal guardian/next of kin, for the publication of any potentially identifiable images or data included in this article.

## Author Contributions

TZ contributed to the conception, design, and radiological expertise, helped to select and assess cases, conducted the data analysis, and drafted and approved the final manuscript. LJ and XM contributed to the drafting and revision of the manuscript. HL and YW offered data collection. SL and GZ contributed radiological expertise and offered a critical review of the manuscript for intellectual content. All authors have read and approved the final manuscript.

## Conflict of Interest

The authors declare that the research was conducted in the absence of any commercial or financial relationships that could be construed as a potential conflict of interest.
